# Editorial: Insights in RNA: 2022

**DOI:** 10.3389/fgene.2024.1382435

**Published:** 2024-02-22

**Authors:** Rui Li, Yadong Zheng, William C. Cho

**Affiliations:** ^1^ Key Laboratory of Applied Technology on Green-Eco-Healthy Animal Husbandry of Zhejiang Province, Zhejiang Provincial Engineering Laboratory for Animal Health Inspection & Internet Technology, Zhejiang International Science and Technology Cooperation Base for Veterinary Medicine and Health Management, China-Australia Joint Laboratory for Animal Health Big Data Analytics, College of Animal Science and Technology and College of Veterinary Medicine of Zhejiang A&F University, Hangzhou, China; ^2^ Department of Clinical Oncology, Queen Elizabeth Hospital, Hong Kong, China

**Keywords:** message RNA, noncoding RNA, microRNA, RNA folding, RNA-seq

RNA is a captivating realm that consists of coding RNA and noncoding RNA, with the latter experiencing significant growth in recent years. The advent of next-generation sequencing technologies has shed light on the functional importance of noncoding RNA, dispelling the notion that it is merely “junk” in the genome. RNA plays a fundamental role in various biological processes, including genetic inheritance, mediating interactions between DNA and proteins, catalyzing biochemical reactions, and regulating gene expression. Given the rapid advancements in this field, we have launched the Research Topic “Insights in RNA: 2022” to provide an overview of the latest technologies, discoveries, and theories in the RNA world, inspiring future research endeavors.

This Research Topic includes a total of 9 published papers, comprising 3 research articles and 6 reviews (https://www.frontiersin.org/research-topics/46100/insights-in-rna-2022/articles). In one of the research articles by Zahid et al., they identified five potential targets of brain-specific microRNA-153 in Alzheimer’s disease (AD). These targets, including ortilin-related receptor 1 (SORL1), amyloid precursor protein (APP), phosphatidylinositol binding clathrin assembly protein (PICALM), upstream stimulatory factor 1 (USF1), and presenilin-1 (PSEN1), are part of a protein interaction network implicated in AD. Previous studies have shown that APP, the precursor of the β-amyloid (Aβ) peptide, is downregulated in AD patients and negatively correlated with miR-153 expression (Liang et al., 2012; Long et al., 2012). AD is characterized by the accumulation of Aβ in senile plaques, and chronic brain hypoperfusion (CBH) has been implicated in Aβ deposition and synaptic plasticity reduction (de la Torre, 2021). In a recent study using a rat model of CBH, miR-153 was found to be upregulated and associated with impaired presynaptic vesicle release. Overexpression of miR-153 led to the suppression of several proteins involved in presynaptic vesicle release. Conversely, knockdown of miR-153 attenuated the decrease in presynaptic vesicle release and cognitive decline in the rat model, suggesting that miR-153 plays a role in impaired presynaptic plasticity in CBH (Yan et al., 2020). Notably, the expression levels of miR-153 in AD patients and the CBH rat model show opposite trends. This discrepancy may be due to differential expression at different stages of AD, with increased expression during synaptic dysfunction, which is implicated in the initiation of AD (Chakroborty et al., 2019). In addition to its role in presynaptic vesicle release, miR-153 has been shown to inhibit the differentiation and proliferation of neural stem cells, which have potential as disease-modifying biologics for AD treatment (Dong et al., 2023). Although the exact role of miR-153 in AD is still being elucidated, it is considered a promising therapeutic target for combating this disease. Furthermore, the paper discusses the therapeutic potential of other miRNAs in AD (Zainal Abidin et al.).

Understanding the intricate structures of RNA is crucial for unraveling its functions, and the field of RNA structure prediction has garnered considerable interest. Machine learning (ML) algorithms have emerged as a potential approach for predicting potential structures of RNA sequences. In this Research Topic, Chasles and Major evaluated the effectiveness of ML algorithms with different parameters in predicting RNA folding, highlighting the need to optimize models for specific data. Various ML methods with different model architectures and output predictions have been developed, such as RNA3DCNN, trRosettaRNA, and DRfold. ML has also been successfully employed in identifying binding sites of metal ions, including Mg^2+^, Na^+^, and K^+^ (Zhao et al., 2023). However, when it comes to truly generalizing ML methods to unseen, structurally distinct RNA families (not just unseen sequences), they do not appear to have an advantage over traditional non-learning techniques (Wu et al., 2023). To further advance the application of ML in RNA structure prediction, it is necessary to establish standardized benchmark training examples/datasets, possibly using a cluster-based k-fold cross-validation approach (Wu et al., 2023).

Sequencing technologies, particularly RNA sequencing (RNA-seq), have revolutionized our understanding of cellular and tissue physiology and pathology. By providing genome-wide RNA expression profiles, transcriptomics enables us to examine the transcriptional landscape and identify differentially expressed molecules relevant to the biology and pathogenesis of interest. However, reliable transcriptomic data necessitates the extraction of high-quality total RNA. In this Research Topic, He et al. conducted a comparative evaluation of the performance of various commercial RNA extraction kits and examined the factors influencing RNA quality in sera used in clinical settings. They observed significant variations in the quality of extracted total RNA when different commercial kits were employed, and identified storage time and temperature of sera as negative factors. Furthermore, they found that all preanalytical processes introduced a bias to the transcriptomes, highlighting the importance of RNA quality control prior to RNA-seq. These findings emphasize the need for ensuring high-quality RNA for accurate and reliable downstream analyses.

The full review papers included in this Research Topic cover a range of important areas in RNA research. These include intron biology, RNA sequencing technologies for T cell receptors, RNA-based therapeutics for the treatment of lung and central nervous diseases, and the mechanisms underlying mRNA deadenylation. These reviews provide readers some of the latest advancements and hot topics in of RNA research, offering valuable insights for future studies and guiding researchers towards new directions.
